# Balance Training in Older Adults Using Exergames: Game Speed and Cognitive Elements Affect How Seniors Play

**DOI:** 10.3389/fspor.2020.00054

**Published:** 2020-05-12

**Authors:** Phillipp Anders, Espen Ingvald Bengtson, Karoline Blix Grønvik, Nina Skjæret-Maroni, Beatrix Vereijken

**Affiliations:** ^1^Department of Neuromedicine and Movement Science, Norwegian University of Science and Technology (NTNU), Trondheim, Norway; ^2^Sunnaas Rehabilitation Hospital, Bjørnemyr, Norway

**Keywords:** balance training, older adults, exergaming, movement characteristics, game settings

## Abstract

Falls in older adults are a serious threat to their health and independence, and a prominent reason for institutionalization. Incorrect weight shifts and poor executive functioning have been identified as important causes for falling. Exergames are increasingly used to train both balance and executive functions in older adults, but it is unknown how game characteristics affect the movements of older adults during exergaming. The aim of this study was to investigate how two key game elements, game speed, and the presence of obstacles, influence movement characteristics in older adults playing a balance training exergame. Fifteen older adults (74 ± 4.4 years) played a step-based balance training exergame, designed specifically for seniors to elicit weight shifts and arm stretches. The task consisted of moving sideways to catch falling grapes and avoid obstacles (falling branches), and of raising the arms to catch stationary chickens that appeared above the avatar. No steps in anterior-posterior direction were required in the game. Participants played the game for eight 2 min trials in total, at two speed settings and with or without obstacles, in a counterbalanced order across participants. A 3D motion capture system was used to capture position data of 22 markers fixed to upper and lower body. Calculated variables included step size, step frequency, single leg support, arm lift frequency, and horizontal trunk displacement. Increased game speed resulted in a decrease in mean single support time, step size, and arm lift frequency, and an increase in cadence, game score, and number of error messages. The presence of obstacles resulted in a decrease in single support ratio, step size, cadence, frequency of arm lifts, and game score. In addition, step size increased from the first to the second trial repetition. These results show that both game speed and the presence of obstacles influence players' movement characteristics, but only some of these effects are considered beneficial for balance training whereas others are detrimental. These findings underscore that an informed approach is necessary when designing exergames so that game settings contribute to rather than hinder eliciting the required movements for effective balance training.

## Introduction

The rapidly aging population in industrialized countries and concurrent strains on the health care system necessitate more cost-effective treatment and prevention options to counteract age-related decline in functioning. Falls in older adults are among the main causes for hospitalization and institutionalization (Kannus et al., 2005) and significantly impact the cost burden on health care budgets worldwide (Heinrich et al., 2010). Furthermore, both actual falls and fear of falling are associated with reduced activity (Yardley and Smith, 2002; Hornyak et al., 2013), which in turn increases the risk for developing chronic conditions.

There is good evidence that balance training reduces fall risk (e.g., Buchner et al., 1997) and can counteract inactivity caused by fear of falling (Gusi et al., 2012). A cost-effective way to administer additional balance training with good adherence rates is exergaming (Burke et al., 2009; Skjæret et al., 2016). Exergames are videogames that require bodily movements as input to play the game (Brox et al., 2011). The term is used both for vigorous exercises and for less intensive exercises such as balance training or seated upper body exercises. Furthermore, exergames allow for home-based training in elderly, as demonstrated in early studies using force-sensitive matbased stepping exergames (Schoene et al., 2011; Smith et al., 2011). In recent years, exergames have gained popularity both as a complementary tool or as a replacement for traditional exercise and rehabilitation (e.g., Mellecker et al., 2013), with the same or better effectiveness compared to usual care (Skjæret et al., 2016). In addition, exergames have been shown to have positive effects on physical activity in general (e.g., Höchsmann et al., 2016; Rhodes et al., 2017), as well as in specific rehabilitation settings (e.g., Laver et al., 2012; Baltaci et al., 2013). Ongoing developments in game technology, such as videobased motion detection of the player, allow for exergames with more variation in movements compared to mat-based exergame systems.

Correctly executed steps are important to maintain balance and to avoid falls (Robinovitch et al., 2013). In daily life, one is often required to make quick, unanticipated steps to react to changing circumstances in order to avoid a fall (Lord and Fitzpatrick, 2001). This requires both the mental and physical capacity to react to an unknown situation. Caetano et al. (2016) showed that the ability to adapt gait is an important factor in predicting fall risk. Furthermore, they showed that concurrent cognitive tasks resulted in older adults reducing their walking speed and shortening their steps during the stepping and avoidance paradigm used in their studies (Caetano et al., 2017, 2018).

Stepping exergames are well suited for creating artificial situations in which unplanned steps are needed and have been shown to reliably assess fall risk in community-dwelling older adults (Schoene et al., 2011). Furthermore, a recent systematic review of randomized control trials about the effect of balance exergames in older adults (Fang et al., 2020) found improvements in functional performance and balance confidence with respect to dynamic balance, perceived balance, chair stand test, and balance test batteries. Non-significant improvements in static balance and proactive balance were speculated to be caused by ceiling effects in the tests used.

Despite the good evidence for the use of stepping exergames to train balance, there is an overall lack of knowledge on the actual movement characteristics that are elicited by exergames, both in elderly players and in other populations. This knowledge is crucial for the development of evidence-based, targeted exergames if the intention is to provide effective training and rehabilitation in the aging population. This is even more critical when exergames are to be used unsupervised in home-based training. To date, most of the research has focused on the effectiveness of, and adherence to, training programs using exergames compared to usual care, not on whether the intended movements are actually elicited. Without knowledge about the movement characteristics elicited during gameplay, it is difficult to design effective balance training exergames or interpret the effects, or lack thereof, of exergaming interventions in clinical trials.

In order to design effective exergames that achieve high adherence rates over longer periods of time, expertise from multiple disciplines is necessary. On the one hand, health care professionals need to contribute with their knowledge about which exercises and movements are required to train specific functions. On the other hand, the expertise of programmers and video game designers is crucial, both for the technical aspects and to ensure that the exergames are enticing and inherently motivating over longer periods of time. Furthermore, to assess whether the intended movements are indeed elicited during gameplay, the expertise of human movement scientists is required. Such multidisciplinary effort at the intersection of health, movement science, and computer science becomes even more crucial given the lack of knowledge about movement characteristics of elderly exergame users that are elicited by different settings and potential additional cognitive challenges within the exergame.

A critical element for training and rehabilitation programs to be effective is adherence to the program. A common strategy to achieve high adherence during exergaming is to try to keep the player in a so-called flow-zone (Csikszentmihalyi, 1975). In the flow-zone, a player is neither overchallenged nor bored by the exergame. One way to achieve this is by dynamically adjusting the game settings during the game to match the performance of the player. For example, game speed can be changed to adjust the difficulty level of the exergame, thereby personalizing the exergame to the player's abilities and enabling progression (e.g., van Diest et al., 2013). However, very little is known about how a change in game speed affects the movement characteristics of the player, and whether these effects are beneficial or detrimental for the desired training or rehabilitation effect. For example, if a higher game speed would lead to less carefully executed movements, as in a speed-accuracy trade-off (cf. Heitz, 2014), this might not be beneficial to achieve the desired training effects. A better understanding of the effects of game speed on elicited movement characteristics is thus necessary to inform the development of targeted exergames for balance training and prevention of functional decline.

Similarly, additional challenges in the form of cognitive elements are used often in exergames to keep the player in the flow-zone or to add cognitive training to the physical exercise (Anders et al., 2018). These additional cognitive elements can be in the form of extra tasks in the exergame such as counting, matching objects, or avoiding obstacles. Cognitive elements in exergames can create a dual task situation in which the player needs to focus on two or more things simultaneously, thereby training cognitive function as well as physical function. Evidence suggests that dual task training can improve walking performance of older adults (Wollesen et al., 2017). However, the effect of additional cognitive elements on the player's movement characteristics in exergames is rarely explored. In a rare exception, Skjæret-Maroni et al. (2016) found a decrease in the quality of movement characteristics needed to train balance when cognitive tasks were present during exergaming, underscoring the need for better knowledge about how game settings influence elicited movements during exergaming.

The objective of the current study is to address these gaps in our knowledge by investigating the effect of game speed and obstacles on movement characteristics in older adults playing a balance training exergame. As there is good evidence that step-based balance training reduces the risk of falls in older adults (e.g., Okubo et al., 2017), we chose a stepping exergame for balance training and assessed the movement characteristics of older adults playing at two different speed settings and either with or without obstacles to avoid. To the best of our knowledge, there is no previous study that assessed the effects of game speed and obstacles in exergames on movement characteristics in older adults. This knowledge is crucial for both health care professionals and developers of exergames in order to choose between existing or design new exergames that are most beneficial for balance training.

We expected that both game speed and the presence of obstacles would influence movement characteristics, and that some of these effects would be beneficial for balance training but others detrimental. Furthermore, in exergames with higher cognitive load, we expected the participants to make smaller steps.

## Methods

### Participants

Fifteen older adults between 65 and 83 years of age participated in an experimental laboratory study at the Norwegian University of Science and Technology (see Table 1 for participant characteristics). To be included, participants had to be 65 years or older and live independently. Participants were excluded if they had an injury or had undergone surgery of the back or the lower extremities during the last 6 months, or if they were unable to follow instructions given by the researchers.

**Table 1:** Participant characteristics.

	**Mean**	**SD**	**Range**
**Age (years)**			
Female (*N* = 7)	72.6	4.7	65–79
Male (*N* = 8)	74.8	4.0	70–83
Total (*N* = 15)	73.7	4.4	65–83
**Height (cm)**			
Female	166.8	3.5	162.8–172.2
Male	175.2	6.1	165.8–183.5
Total	171.3	4.1	162.8–183.5
**Weight (kg)**			
Female	65.4	4.9	60–74.4
Male	76	4.1	70.2–82.8
Total	71	7	60–82.2

The protocol was approved by the Regional Committees for Medical and Health Research Ethics, Norway. All participants gave written informed consent before data collection in accordance with the Declaration of Helsinki.

### Procedure

**Figure 1:**
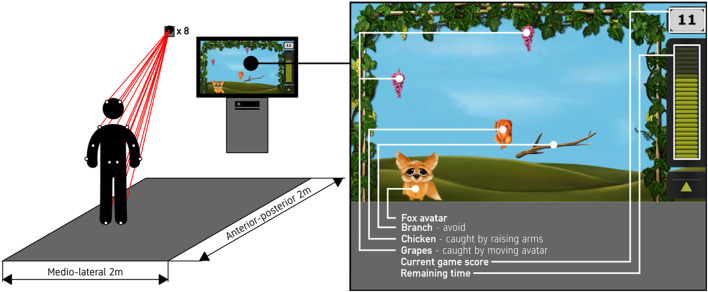
Graphical image of the exergame area (left panel) and a screenshot of “The Fox” exergame (right panel) showing all exergame elements (Fox-avatar, grapes, chickens, branches, game score, and remaining game time).

All participants played a step-based balance training exergame (“The Fox,” SilverfitBV, the Netherlands). The aim of the exergame was to feed a fox-avatar by moving it sideways into the trajectory of falling grapes. The avatar mirrored the lateral movements of the participants, so when the participants placed themselves to the far right of the exergaming area, the avatar would be on the far-right side of the screen. The exergaming area was set to 2 by 2 m as shown in Figure 1. Thus, the movement of the avatar from one side of the screen to the opposite side corresponded to a 2 m distance in the physical playing area. The game itself did not determine any required step size, as participants can take several smaller or fewer larger steps to move the avatar the desired distance. Occasionally appearing stationary chickens above the fox-avatar were caught by raising at least one arm, which triggered the fox to jump. Only arm movements that resulted in at least one hand being lifted higher than the head were accepted by the exergame as a trigger for a jump. Other arm movements, e.g. to maintain balance, were ignored by the game. Participants had a time window of 7 s to place themselves under a chicken and raise at least one arm to trigger a jump, before the prey disappeared again.

No movement in the anterior-posterior direction was required to play the exergame. The exergame system used a Microsoft Kinect v2 camera (Microsoft Corporation, Redmond, WA, USA) to capture the movements of the participants. The Kinect v2 was used as input for the exergame only and not for data capturing.

Each participant played eight 2-min exergame trials at two speed settings and either with or without additional obstacles, falling branches, that had to be avoided. The width of a falling branch can be seen in Figure 1. The distance between the grapevines on either side corresponded to a physical distance of 2 m, a branch covered approximately 57 cm (35%). Increasing the exergame speed led to an increased number of grapes and branches falling simultaneously on the screen (from 1–3 to 3–5 grapes, and from 1–2 to 1–3 branches) and reducing their time on the screen from 8–10 s to 6–9 s (measured from top to bottom of the screen for missed grapes and branches). Neither the number of chickens nor their time on the screen were affected by changes of the game speed. The four conditions were played twice in counterbalanced order across participants. Participants had a 2-min break between exergame trials. Before the first trial, each participant tested the game at a lower speed setting to ensure that they understood the task and that they were able to perform all required movements. After the participants completed all eight trials, they performed a range-of-motion test to quantify that they were able to perform all required movements such as arm lifts and forward and sideways steps.

To record the participant's movements throughout the trials, 22 retroreflective markers were placed on anatomical landmarks using double-sided tape and a headband. Markers were placed bilaterally on the posterior and anterior side of the head, ulna styloid process, lateral epicondyle of the humerus, acromion, ilium posterior superior, ilium anterior superior, femur lateral epicondyle, lateral malleolus, and lateral distal phalange 1 (big toe), as well as one marker on the sternum and one on the center of the right thigh. A 3D motion capture system (OQUS, Qualisys, Gothenburg, Sweden) consisting of eight cameras was used to record the spatial positions of the markers throughout all exergame trials, with a measurement frequency of 120 Hz. The cameras were wall-mounted to minimize blockage by the TV screen or the Kinect v2 camera. Markers that fell off during a trial were replaced before the start of the next trial.

The output of the exergame on the 55” (139.7 cm) TV screen was captured using Open Broadcaster Software Studio (version 21.0; Open Broadcaster Software Studio, 2018). Figure 1 shows an exemplary frame including all game elements. The game score displayed on the top right of the screen was explained to the participants, but they were not specifically encouraged to achieve the highest possible score.

If the participant moved outside the predefined exergaming area of 2 by 2 m, the game stopped and an error message appeared on the screen containing information on how to resolve the issue, e.g., “You are too far in the back. Please move closer to the screen.” The exergame continued as soon as the participant returned to the exergaming area. The size of the exergaming area was not indicated on the floor in order to mimic a home-based setting.

### Data Analysis

A custom Matlab script (version: 9.3.0, Mathworks Inc., Nantick, MA) was used to analyze the movement of the extremities as well as the position of the trunk based on the 3D motion capture data. The start and stop of each step was identified using the same method as in Skjæret-Maroni et al. (2016), which used the position of the markers on the lateral malleolus. A step was defined as a ≥ 0.03 m displacement of the toe marker lasting for ≥ 0.05 s. A marker velocity of 0 ± 0.1 ms^−1^ was used for the identification of step initiation and termination. From these, mean duration of single leg support, mean step size, and cadence were calculated for each foot. We chose the term step size rather than step length or step width to capture both sideways and forward aspects of steps, as some participants partly rotated their upper body whereas others remained parallel to the screen while taking steps. Furthermore, the ratio of the total duration of single-leg support (total accumulated single-leg support time divided by trial time) was calculated. Movement data for the arms was used to calculate the number of arm lifts per minute. The start and end of an arm lift was determined by the relationship between the vertical positions of the average height of all head markers compared to the left and right markers on the ulna styloid processes (wrists). The player's position on the playing field was determined by the position of the marker on the sternum. Displacements of the sternum marker were used to create heatmaps that reflected upper body positions of the participants in relation to the screen.

There were slight variations in the duration of each exergame across trials and participants due to error messages that appeared on the screen when a participant left the exergaming area, which temporarily stopped the game but not the 3D motion capture recording. Therefore, we report the ratio for single-leg support and the frequency of arm lifts rather than the total duration of single-leg support and the total number of arm lifts.

Finally, optical character recognition was used on the captured screen frames to identify error messages during each trial, as well as the total game score and the number of chickens caught and branches hit (if present) at the end of each trial.

### Statistics

A linear mixed effect analysis in R (R Core Team, 2019) was used to analyze the effects of game speed, the presence or absence of obstacles, gender, and trial repetition, which served as fixed effects on the investigated movement characteristics, using the lme4 package (Bates et al., 2015). Random effects included intercepts for participants as well as by-participant random slope for the effect of body side (right or left arm or foot). Visual inspection of residual plots did not reveal deviations from normality or homoscedasticity. The computation of p-values was based on conditional F-tests with Kenward-Roger approximation for the degrees of freedom (Halekoh and Højsgaard, 2014). Statistical significance was set at *p* < 0.05. The linear mixed effect analysis was chosen to remove individual differences between participants (Schoene et al., 2011) and to account for the slight gender imbalance without losing statistical power.

## Results

### Game Score

The game score that appeared on the screen after each trial was a combination of grapes and chickens caught and branches avoided (+ 1 point for each grape caught, + 3 points for each chicken caught, and −2 points for each branch hit). The number of grapes presented varied across trials, depending on the chosen game speed, thereby resulting in more opportunities for catching grapes in games with high game speed. In contrast, the number of chickens did not vary with game speed but was constant at 11 chickens per trial. The game score results are shown in Table 2. Not surprisingly, the mean game score was higher in the trials at high game speed without obstacles. The presence of obstacles reduced the game score by approximately 20 points for either game speed setting, while the game score was about 20 points higher in games at high speed compared to games at low speed. The percentage of grapes caught was ~20% higher in low-speed compared to high-speed games. Interestingly, the percentage of grapes caught with or without obstacles was similar, with a small trend to increase when obstacles were present. In contrast, a clear decrease was seen in the percentage of chickens caught in games with obstacles present. Percentage of branches unsuccessfully avoided was very low and slightly higher in games with low speed settings.

**Table 2:** Mean game score and mean percentage of caught grapes, caught chickens, and unsuccessfully avoided branches for all combinations of game speeds and obstacles.

			**Speed**		
			***low***	***high***	**Overall**
Game score	**Obstacles**	*without*	69.8	86	**78.5**
		*with*	48.7	67.4	**58.3**
		**Overall**	**59**	**77.1**	**68.5**
Grapes caught		*without*	77.6%	53.7%	**64.8%**
		*with*	79.8%	59.1%	**69.3%**
		**Overall**	**78.7%**	**56.3%**	**67%**
Chickens caught		*without*	91.3%	93.1%	**92.3%**
		*with*	74.8%	76.1%	**75.5%**
		**Overall**	**82.9%**	**84.9%**	**83.9%**
Branches hit		*with*	5%	3.8%	**4.4%**

*The values in bold font represent the average across game speed, presence of obstacles and overall*.

### Movement Characteristics

#### Single Leg Support

**Figure 2:**
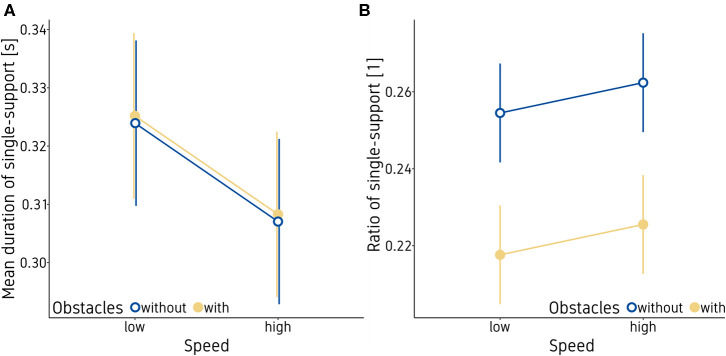
Mean duration of single support events **(A)** and ratio of single support **(B)**. Vertical bars indicate the standard error of the model.

As standing on one leg is an important strategy to train balance, we investigated both the mean duration of single support events per foot (in seconds) and the ratio of single leg support (accumulated single support time during a trial divided by trial time). The mixed effects model indicated that the mean duration of single support events decreased significantly (*p* < 0.001) with an increase in game speed, whereas the ratio of single support increased slightly but not significantly (*p* = 0.189) (see Figure 2 and Table 3). This indicates that participants stood slightly more often but significantly shorter on one foot when playing at higher speed. Furthermore, the ratio of single support decreased when obstacles were present, indicating that participants accumulated less single support time during a trial when having to avoid obstacles. Neither ratio nor duration of single support were significantly affected by trial repetition, gender, or body side (all p's > 0.10).

**Table 3:** The degrees of freedom (df), confidence intervals (CI), and significance level (p) for the mean duration of single support events, ratio of single support, mean step length, cadence, and the frequency of arm lifts.

	**Mean duration of single support (s)**			**Ratio of single support (1)**			**Mean step size (mm)**			**Cadence (min**^****−1****^**)**			**Frequency of arm lifts (min**^****−1****^**)**		
	**df**	**CI**	***p***	**df**	**CI**	***p***	**df**	**CI**	***p***	**df**	**CI**	***p***	**df**	**CI**	***p***
Speed	229.07	−0.03 to −0.01	**<0.001**	229.09	−0.00 to 0.02	0.189	293.14	−13.87 to −0.45	**0.038**	229.07	5.22 to 8.56	**<0.001**	229.07	−2.27 to −0.99	**<0.001**
Obstacles	229.07	−0.01 to 0.01	0.774	229.09	−0.05 to −0.03	**<0.001**	−7.16	−18.54 to −5.12	**0.001**	229.07	−10.97 to −7.63	**<0.001**	229.07	−1.70 to −0.42	**0.001**
Trial	229.07	−0.01 to 0.01	0.582	229.09	−0.01 to 0.02	0.318	−11.83	5.78 to 19.20	**<0.001**	229.07	−0.80 to 2.55	0.306	229.07	−0.97 to 0.32	0.321
Gender	17.31	−0.05 to 0.06	0.909	17.29	−0.02 to 0.07	0.280	12.49	−42.63 to 3.39	0.113	17.31	−2.12 to 14.10	0.166	17.31	−5.64 to 1.94	0.352
Side	229.07	−0.00 to 0.02	0.102	229.09	−0.01 to 0.01	0.982	−19.62	−7.02 to 6.40	0.928	229.07	−0.64 to 2.70	0.229	229.07	−1.20 to 0.08	0.086

*The values in bold font represent significant p values*.

#### Mean Step Size and Cadence

**Figure 3:**
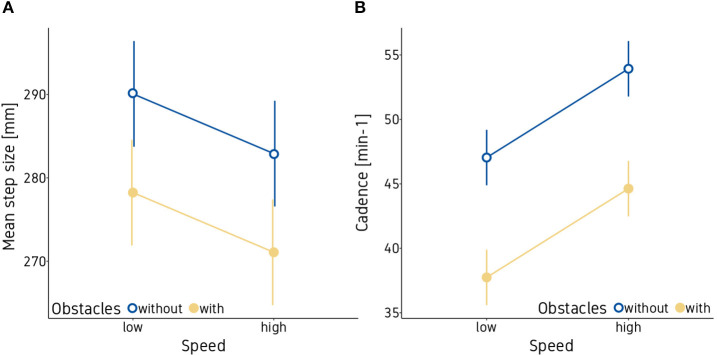
Mean step length **(A)** and cadence **(B)**. Vertical bars indicate the standard error of the model.

Step size and cadence where both affected significantly by the game speed (mean step size: *p* < 0.05; cadence: *p* < 0.001**)** and the presence of obstacles (mean step size: *p* = 0.001; cadence: *p* < 0.001**)**. As can be seen in Figure 3, higher game speed and the presence of obstacles led to a decrease in mean step size (panel A). Cadence decreased as well when additional obstacles were present but increased with an increase in game speed (panel B). In addition, trial repetition had a significant effect on mean step size (*p* < 0.001), with larger steps when participants played the same condition for the second time. See Table 3 for all statistical results for mean step size and cadence.

#### Frequency of Arm Lifts

**Figure 4:**
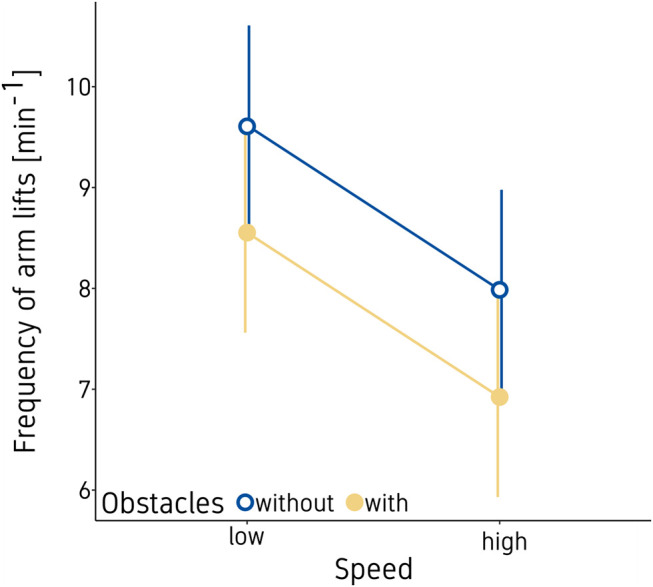
Mean frequency of arm lifts. Vertical bars indicate the standard error of the model.

The frequency of arm lifts decreased with high game speed (*p* < 0.001) and when additional obstacles were present (*p* = 0.001), as shown in Figure 4. Trial repetition, gender, and body side had no significant effects on the frequency of arm lifts (all *p*‘s > 0.08). See Table 3 for all statistical results.

### Player's Position

#### Heatmaps

**Figure 5:**
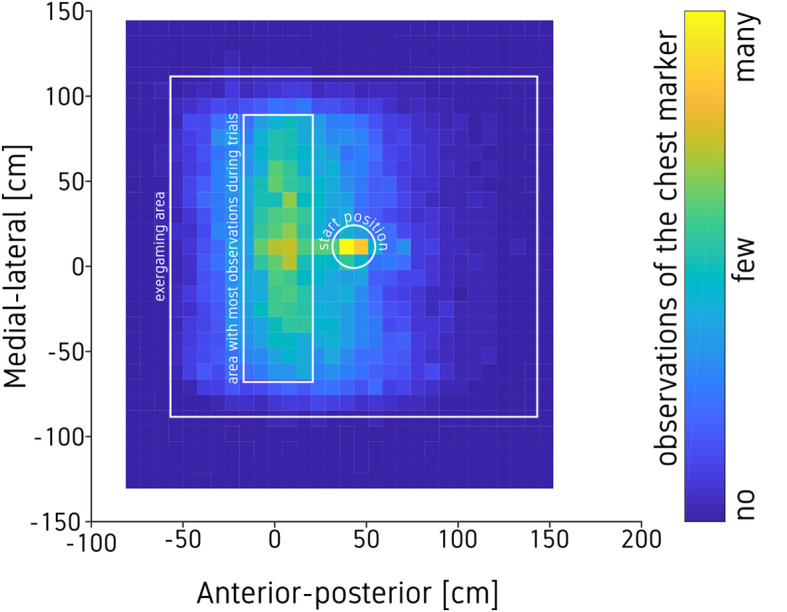
Heatmap of the players' positions in the exergaming area based on the marker on the chest across all conditions and participants. The white square indicates the 2 by 2m active exergaming area. All participants started each trial in the white circle. The white rectangle indicates the area with most observations. The game screen was positioned to the left of the playing area.

As described above, the active area in which the exergame could be played was ~2 by 2 m. The heatmap (see Figure 5) shows the number of observations of the sternum marker in the exergaming area (white square) across all participants and all trials, on a 30 by 30 grid. Warmer colors indicate more observations. The starting position was centered in front of the screen, 0 mm in medial-lateral and 500 mm in posterior direction (white circle in Figure 5). On average, participants drifted ~0.5 m closer to the screen throughout an exergame trial, as indicated by the green and yellow squares, indicating more observations of the sternum marker parallel to the screen (white rectangle). There were no systematic differences in the heatmaps between the different conditions.

#### Moving Outside the Exergaming Area

When a participant moved outside the active exergame area of 2 by 2 m in any direction while playing, an error message would appear on the screen to inform the participant to correct their position in the playing area. We used a custom Matlab script including optical character recognition to detect error messages in the screen recording. Out of 15 participants, only two participants never drifted outside the active exergame area while playing the exergames. The remaining participants moved outside the area between one and nine times across all conditions. On average, 0.38 error messages per exergame trial were observed across all exergame trials that had valid screen captures (113 out of 120 trials). The majority of error messages were triggered by participants who drifted too close to the screen while playing (36 out of 43 error messages in total). The average time to resolve this was 2.44 s. The remaining error messages were divided between being too far to the left (4 times, on average 1.34 s to resolve), too far to the right (once, 0.67 s), and too far back (twice, 1.70 s).

More error messages were triggered in trials at high game speed compared to those at low game speed (see Table 4). However, a chi-square test indicated that the presence or absence of obstacles showed no consistent effect on the number of error messages triggered, nor was there a significant interaction between game speed and the presence of obstacles, $\chi_{\left(1\right)}^{2}$ = 1.203, *p* > 0.05.

**Table 4:** Error messages triggered by the players moving outside the active exergaming area for each combination of settings.

**Error messages**		**Obstacles**		
		***without***	***with***	**Total**
**Speed**	*low*	5	8	13
	*high*	17	13	30
	**Total**	22	21	43

## Discussion

The purpose of this study was to investigate the effect of game speed and obstacles on movement characteristics of older adults who played a step-based balance training exergame. According to a meta-analysis by Sherrington et al. (2011), effective balance exercises should include displacement of the center of gravity and reduced base of support. Therefore, we assessed mean step size and cadence, the ratio and duration of single-leg support, as well as the frequency of arm lifts as arm lifts and stretches influence the center of gravity. Both changes in game speed and additional cognitive elements are used regularly in exergames to keep the player in the flow-zone and/or train cognitive and physical functions simultaneously. However, as the results of the current study show, changing the settings of an exergame can have both positive and negative effects on the player's movement characteristics with respect to training balance. Below, we discuss our main findings regarding the effects of game speed, the presence of obstacles, and trial repetition on movement characteristics, the consequences of these effects for balance training, and their relevance for choosing existing or developing new exergames for balance training in older adults.

### Game Speed

Higher game speed led to shorter single support events, shorter steps, fewer arm lifts, and increased cadence. Higher cadence is associated with more frequent weight shifts, which are considered beneficial for effective balance training (Sherrington et al., 2011). Furthermore, eliciting faster steps during gaming at higher speed may benefit the training of a quick step to avoid, or recover from, a balance disturbance and imminent fall. On the other hand, participants were more likely to move outside the active exergaming area when playing at higher speed, as indicated by more than twice as many error messages. This would cause the exergame to stop, thereby potentially interrupting the participant's flow. Further research is necessary to find an optimal balance between these contrasting effects of game speed when designing or choosing the most beneficial game and game settings for balance training. This is further attested to by our results regarding game score. As higher game speed led to higher scores, this may have important ramifications for exergames where achieving a high score is the focus for the player, as game speed affects the movement characteristics as well.

### Obstacles

The presence of obstacles led to shorter steps, reduced cadence, fewer arm lifts, and a decrease in the ratio of single-support time. A likely explanation for these changes in movement characteristics is increased cognitive load caused by the presence of additional exergame elements and potentially conflicting demands on the player. These results indicate that although cognitive elements are often added to the game to increase enjoyment and/or simultaneously train cognitive functions, they can lead to unwanted side effects on elicited movement characteristics that are less favorable for balance training (see also Skjæret-Maroni et al., 2016).

Furthermore, we observed that when participants moved their avatar toward the edge of the screen, the avatar was occasionally trapped there by falling branches that blocked the path back to the middle or the other side of the screen. This resulted in participants waiting for the obstacle to pass before continuing to play the game and try to catch grapes and chickens. The waiting period in which the participants stood still could take up to several seconds. The low percentage of collisions with branches indicates that participants indeed tried to avoid being hit by branches as much as possible, even when that meant having to wait for the branch to pass before continuing to catch chickens and grapes. Although having to avoid obstacles may be positive in terms of adding cognitive training and enjoyment to the exergame, this may also lead to strategies and movements that are considered less beneficial for the training of balance. Waiting for a situation to resolve should be avoided by the game design, for example by allowing the avatar to move forward or backward around the obstacle. An alternative solution could be to introduce e.g., an extra bracing position to protect the avatar from falling branches which could simultaneously challenge balance. Our results underscore that the effects of additional cognitive elements on intended movement characteristics need to be taken into account when designing or using exergames for balance training.

### Trial Repetition

Participants played each condition twice, but we did not expect to find learning or fatigue effects with such short exposure. Although most movement characteristics were indeed unaffected by the repetition, we did find larger steps on the second trial compared to the first. A possible explanation for this might be that in the second trial, participants were more familiar and comfortable with playing the exergame in general and the movements required to play the exergame in particular. Pure sideways stepping without a forward component to the movement is less common in everyday life. Therefore, it may be speculated whether participants needed a short adaption phase in order to perform this movement with enough confidence to perform larger steps. In that respect, the observed increase in mean step size from trial one to trial two is a positive result, as stepping sideways can be an important strategy to recover postural control after a balance disturbance (Hsiao-Wecksler and Robinovitch, 2007). Furthermore, this result indicates also that exergames might have an immediate short-term learning effect.

### Other Observations and Lessons Learned

Although the exergame itself did not distinguish between different ways of raising arms to catch chickens, we observed several distinct playing styles. Some participants raised both arms to catch chickens, whereas others raised one arm only. One participant did not raise the arms at all during the game and did not catch any chickens. These differences did not result from physical limitations, as we checked that all participants were able to perform the required movements with both arms. Some participants also used the jump of the avatar to catch falling grapes faster instead of waiting underneath the trajectory of the falling grapes. These observations indicate that exergame players may perform or play in many different ways that can benefit or hinder the intended balance training. Ideally, the game technology used should be able to distinguish correct vs. incorrect movements in these situations and provide feedback in order for the exergame to function as an effective balance training and rehabilitation tool (cf. Vonstad et al., 2018). Thus, the knowledge gained by investigating different movement characteristics displayed by the players when exergaming should be used in the design and development of new exergames for health benefits.

Throughout each trial, participants moved ~0.5 m closer to the screen on average. There was no incentive in the exergame to do so as the game was played on a two-dimensional plane parallel to the screen. This gradual forward movement was observed across all conditions and participants. The implemented elements in the game such as the falling branches did not affect this behavior. A possible explanation for the drift toward the screen might be that a pure sideways movement is difficult to achieve and less common in activities of daily living compared to side steps with an additional forwards component to them, as in avoiding an obstacle in the path of progression. Alternatively, it can be speculated that the older adults who served as participants in this study struggled to learn to use the system quickly enough to avoid drifting outside the exergaming area. Younger adults and children, the main customer-base of video game technology, grew up using technology such as Microsoft's Kinect or Sony's EyeToy and are therefore more familiar with digital avatars mirroring movements, as well as with the inherent limitations caused by the limited field-of-view of the infrared cameras. Thus, they may be more likely to reposition themselves when they drift away from the center of the observable area. Although we did not mark the exergaming area with tape in order to mimic a more natural home-based setting, proper delimitation might help to reduce the ambiguity concerning one's position within the exergaming area.

Over time, the accumulation of the drift forward resulted in error messages and breaks in the game until participants stepped back into the active exergame area. Though usually of short duration and easily corrected by the player, those error messages and game breaks likely result in a disruption of the flow-zone (Csikszentmihalyi, 1975), with associated potential negative consequences for enjoyment and adherence. However, as we did not collect data on enjoyment of the exergame, this remains an assumption that needs corroboration in further studies. Yet, future development of exergames could take our findings into account by letting the game stimulate the player to move back toward the center of the play area without disrupting the exergame experience by error messages and game breaks.

The current study was designed to observe immediate effects of game settings on movement characteristics in a single exergaming session. But even across only two trial repetitions, steps became significantly larger. In order to achieve lasting improvements in the ability to maintain balance, multiple sessions over a longer period of time are needed. With more repetitions, people who routinely use exergames for balance training might show additional changes in movement characteristics in reaction to changes in game speed or additional challenges. Further research is needed with longer follow-up time to study potential effects over extended playing time.

### Limitations

The current study offers several insights into how game settings in exergames influence the movement characteristics of older adults, allowing to provide recommendations for the development and usage of exergames to elicit the movements necessary to train balance in older adults. However, a few limitations should be highlighted as well. First, this study was conducted using a screen-based exergame. Newly developed exergames might use newer technologies such as immersive virtual reality or gamified objects in the environment rather than TV screens. However, we believe that many of the lessons learned may be transferrable to virtual reality exergames, since the basic concepts for eliciting the desired vs. less desirable movements may largely be the same. Furthermore, older adults are a specific subgroup of users who, at the moment, might be less familiar with modern game and simulation technologies than younger generations. However, continued developments in non-immersive virtual reality and increasing tech-savviness of new generations of older adults may contribute to the accessibility of exergame technology for a wider audience. Finally, although no formal tests of the participants' physical and mental capacity were performed in this study, all participants were healthy for their age and living independently. Further research should broaden out to participants with a wider range of functional capacities to investigate how they would react to changes in game speed and additional challenges.

### Next Steps

Methods to deliver safe, home-based, low-cost balance training to older adults in order to prevent falls and to maintain independence are an important issue for future research, and the availability of a control system implemented in an exergame that maximizes the likelihood of eliciting the most beneficial balance-training movements is of great interest. There are still several unanswered questions, such as potential changes in movement characteristics when playing over extended periods of time and to what extent comparable findings would be produced using different game settings or different exergames. Furthermore, personalizing the delivery of exergame training to the abilities and preferences of older adults, including providing feedback about the correctness of performance, might help accommodate differences in functioning in this heterogenous age group and potentially lead to additional improvements in balance training outcomes.

## Conclusion

Together, the results of the current study provide important insights into how the settings of an exergame influence the movement characteristics of older adults when playing a step-based balance training exergame. Higher game speed led to faster-paced movements with shorter duration of single support, whereas additional cognitive elements in the form of obstacles to avoid led to slower movements and smaller steps. Some of these effects on movement characteristics are beneficial for balance training, whereas others likely make the exergame less efficient. Therefore, informed decisions are necessary when designing new, or choosing between existing, balance training exergames if they are to be effective tools for balance training in older adults.

## Data Availability Statement

The datasets generated for this study will not be made publicly available. Due to Norwegian legislation, the dataset for this article is not open access. Questions regarding the dataset can be sent to Beatrix Vereijken, beatrix.vereijken@ntnu.no.

## Ethics Statement

The studies involving human participants were reviewed and approved by Regional Committees for Medical and Health Research Ethics. The patients/participants provided their written informed consent to participate in this study.

## Author Contributions

All authors contributed to the conception and design of the study. PA, EB, and KG collected the data. PA and EB processed the data. PA performed statistical analyses. PA and BV drafted the manuscript. All authors contributed to manuscript revision, read, and approved the final version.

## Conflict of Interest

The authors declare that the research was conducted in the absence of any commercial or financial relationships that could be construed as a potential conflict of interest.
